# Clinical Behavior in Metastatic Brain Disease Is Not Influenced by the Immunological Defense Mediated by CD57^+^ NK-Cells

**DOI:** 10.1155/2012/834852

**Published:** 2011-07-14

**Authors:** J. Vaquero, M. Zurita, S. Coca

**Affiliations:** ^1^Laboratory of Experimental Neuro-Oncology, Neuroscience Research Unit, Puerta de Hierro Hospital, Autonomous University, 28222 Madrid, Spain; ^2^Neurosurgical Service, Puerta de Hierro Hospital, Autonomous University, 28222 Madrid, Spain; ^3^Department of Pathology, Central Defense Hospital, 28047 Madrid, Spain

## Abstract

*Objectives*. The purpose of the present study is to verify if the degree of immunological response against metastatic tumors, measured by the number of CD57^+^ NK-cells in the tissue of a brain metastasis, influences the later development of new brain metastases or tumor recurrence. *Patients and Methods*. CD57^+^ NK-cells were immunohistochemically identified in the resected tumor, in a series of twenty patients operated on by a single brain metastasis secondary to lung adenocarcinoma. In each case, the degree of CD57^+^ NK-cells infiltration within the tumor tissue and the period free of new intracranial disease after brain surgery were recorded. *Results*. All the studied tumors showed variable number of CD57^+^ NK-cells (mean ± standard deviation: 8.4 ± 4.8 per microscopical field, at 200x). The period free of intracranial disease ranged between 10 and 52 weeks (mean ± standard deviation: 22.7 ± 11.9). Statistical analysis showed that there was no correlation between the degree of NK-cells infiltration within the resected tumor and the period free of intracranial disease after surgery (*P* > 0.05). *Conclusion*. This finding supports that clinical behavior in metastatic brain disease is not influenced by the immunological response mediated by CD57^+^ NK-cells.

## 1. Introduction

At present, it is accepted that Natural Killer (NK) cells are a subset of lymphocytes with an important role in the early response to tumors [1, 2]. They destroy tumor cells by two main cytotoxic pathways: a perforin/granzyme-mediated secretory mechanism and a TNF-family ligand-mediated apoptotic killing. While the former mechanism acts mainly against cultured leukemia cell targets, the second is the way for which NK-cells act against most tumor cell targets [3–6]. Furthermore, the finding that the number of intratumoral CD57^+^ NK-cells influences the survival of the patients has been described for patients with gastric carcinoma [7] or squamous lung cancer [8] supporting the biological effect of immunological defense mechanism mediated by NK-cells. On the other hand, it is well-known that immunologic manipulation, in an early phase of carcinogenesis, can modulate the tumor development [9]. 

Since NK-cells represent a defense against tumors, it is logical to suppose that the capacity to act against tumors through NK-cells will determine the biggest or smaller easiness with which a tumor developed. Therefore, if a patient shows strong immunological defense mechanisms by means of NK-cells in the tissue of a brain metastasis, it is logical to suppose that the tumor recurrence or the development of a new cerebral metastasis will be more difficult. 

In a previous study, we concluded that the number of CD57^+^ NK-cells in the tumor stroma of brain metastases does not correlate with the number of apoptotic tumor cells. This finding suggests that, in brain metastases, apoptosis related to immune response is mainly mediated by activated tumor-infiltrating mononuclear cells other than CD57^+^ NK-cells [10]. We studied here if the number of CD57^+^ NK-cells within the tumor tissue of brain metastases influences the clinical behavior, in terms of influencing the capacity of the brain to be protected for the development of a new metastasis or tumor recurrence.

## 2. Materials and Methods

For this study we selected twenty male patients operated on because of a single cerebral metastasis from lung adenocarcinoma and that developed local recurrence or new brain metastases after surgery. In all cases tumor resection was considered complete, and the patients received holocraneal radiotherapy (30–40 Gy in 10–20 fractions) after surgery. The age at time of surgery ranged between 42 and 78 years (mean: 64 years). 

Paraffin-embedded specimens from the resected tumors were studied. A first study (unpublished data) showed that in all resected tumors, a variable number of cells expressed CD95 (Fas/APO1). From each tumor, a histological slice was processed by haematoxylin-eosin (HE) technique for studying both the histological pattern of the tumor and the degree and distribution of lymphocytic infiltration. Another adjacent histological slice was processed for immunohistochemical expression of CD57. In brief, histological sections from paraffin-embedded samples were mounted on glass slides and were deparaffinized by treatment in xylene for 15 minutes. The sections were rehydrated in a graded ethanol series and rinsed in phosphate-buffered saline (PBS) at pH 7.4. Then, sections were trypsinized for 15 minutes and rinsed in PBS. The slides were then washed in citrate-buffered solution (pH 6.0) for 10 minutes on microwave and then placed in hydrogen peroxide 3% in methanol for 15 minutes in order to block endogenous peroxidase activity, and the sections were immersed in PBS. For detection of NK-cells, primary monoclonal antibody to CD57 (1 : 100, Master Diagnostica, Granada, Spain) was used. Monoclonal antibody was added overnight at 4°C on wet chamber, and the histological slices were again rinsed in PBS. A 30-minute incubation with biotinyled secondary antibody at 37°C was followed by a standard PBS rinse. Another 30-minute incubation with streptavidin-peroxidase complex, at 37°C, was carried out, and then chromogen solution was added (diaminobenzidine). After it, the slices were stained with hematoxylin, mounted, and examined microscopically. In all cases, negative controls were performed using rabbit normal serum as primary antibody. For each tumor, the number of CD57-immunostained cells was counted at least on 10 randomized histological fields, at 200x, and then averaged. In all cases, the evaluation of the number of NK-cells per field was conducted by two investigators with no previous knowledge of the case from each sample obtained. Generally a high grade of agreement between the observers was obtained, but, in any case, the means of values recorded by these investigators were recorded as final values.

## 3. Results

**Figure 1:**
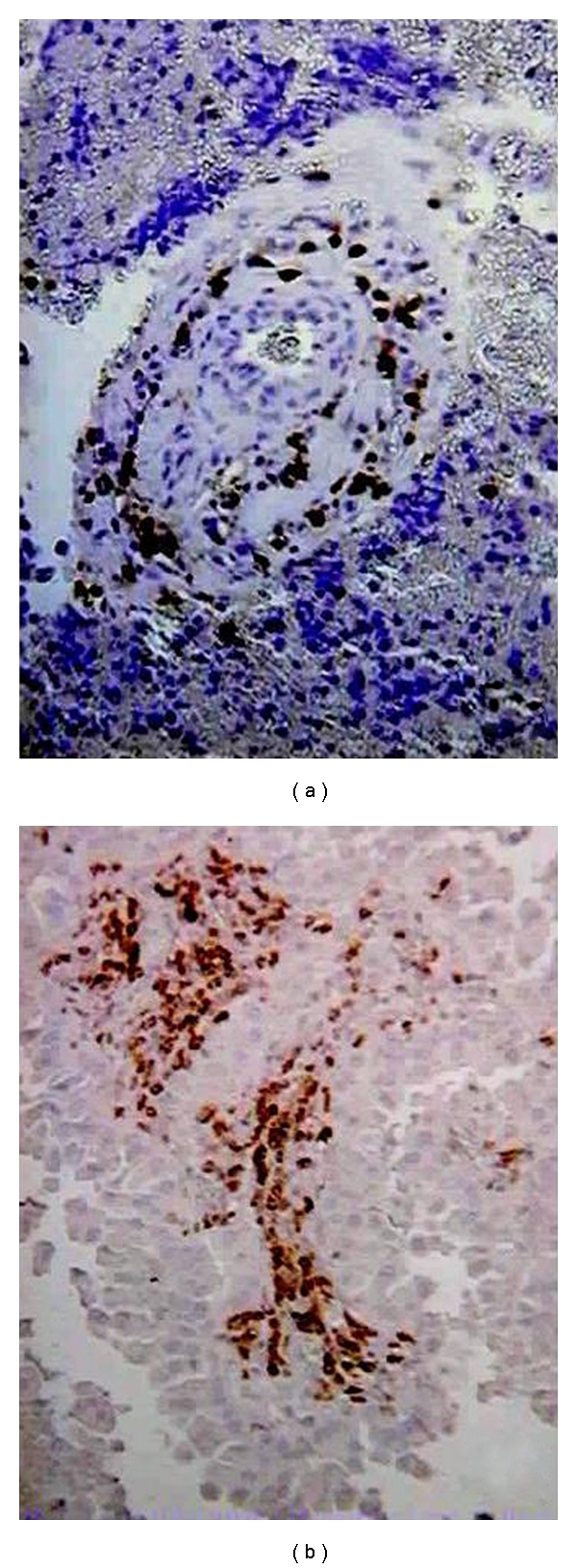
Examples of CD57^+^ NK-cells in the resected brain metastases of the series. NK-cells were generally distributed surrounding the vessels (a) and within the tumor stroma (b). Immunostained CD57^+^ NK-cells can be seen (×200).

All the tumors of the series showed variable number of CD57^+^ NK-cells. These cells did not show a uniform distribution in the tumor but were usually grouped around blood vessels or within the tumor stroma (Figure 1). 

**Figure 2:**
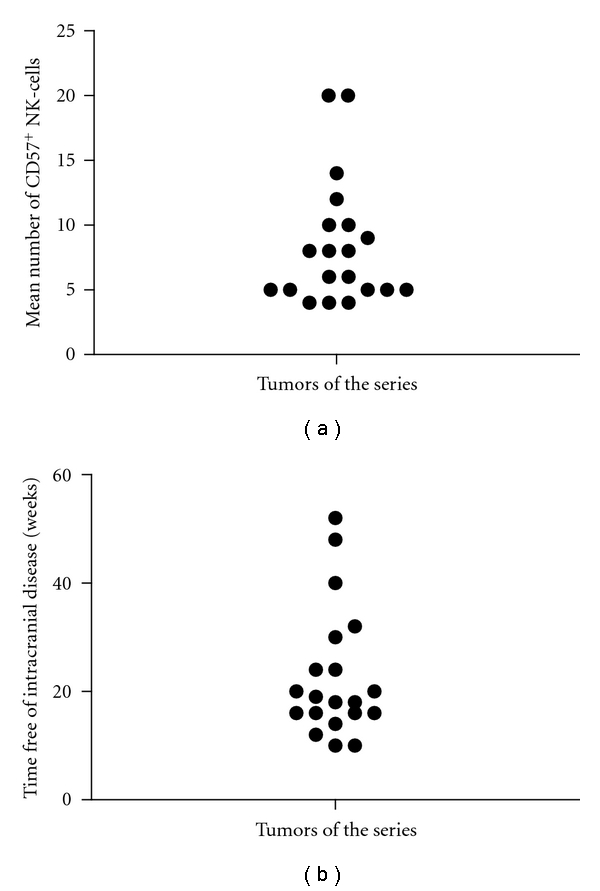
(a) Distribution of the mean values of CD57^+^ NK-cells by microscopical field, at 200x, in the tumors of the series. (b) Time free of local recurrence or new intracranial metastases, in the cases of the series.

After a randomized study of different fields from each tumor, at 200x, a number of 8.4 ± 4.8 (mean ± standard deviation) CD57^+^ NK-cells was estimated as an averaged value (Figure 2(a)) in the series. On the other hand, the period of time free of new cerebral metastases or local tumor recurrence in the patients of the series ranged between 10 and 52 weeks (mean ± standard deviation: 22.7 ± 11.9) (Figure 2(b)).

**Figure 3:**
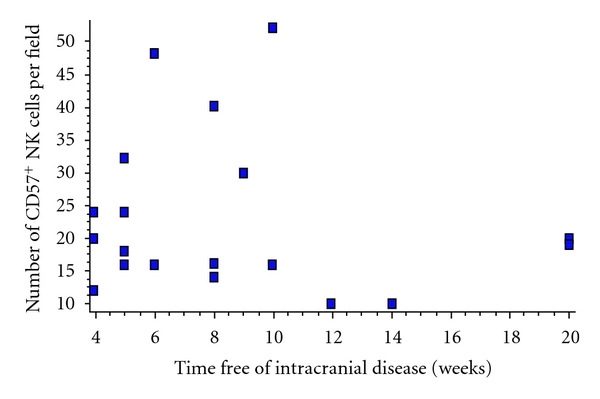
Correlation between the degree of CD57^+^ NK-cells in the resected metastasis and the time free of new intracranial metastatic disease in the cases of the series. Correlation was not found (*P* = 0.63; correlation coefficient (*r*): −0.1128; 95% confidence interval (CI): −0.52 to 0.34).

Lastly, we analyzed for each case the correlation between the time free of local recurrence or new intracranial mestastases after surgery and the degree of CD57^+^ NK-cells infiltrating the resected brain metastasis. After this analysis, a correlation between these two variables could not be found (*P* = 0.63; correlation coefficient (*r*): −0.1128; 95% confidence interval: −0.52 to 0.34) (Figure 3).

## 4. Discussion

For the present study, we have identified CD57^+^ NK-cells in the tissue of resected brain metastases, in a homogeneous series of metastatic brain tumors developed as a result of the spread of a lung adenocarcinoma. Furthermore, in each case, we have recorded the time free of local tumor recurrence or appearance of new cerebral metastases after treatment. Although cytotoxic efficacy of NK-cells in tumor tissue may not be judged by their numerical presence, it is accepted that these cells play an important role in the immunological defense against tumor cells. Thus, it seems logical to assume a relationship between the degree of NK-cells infiltration in tumor tissue and the effectiveness of this type of immunological defense. 

On the other hand, it is accepted that the main mechanism for which NK-cells act is through inducing apoptosis in the tumor cells [3–6], and we previously obtained in all the tumors of the series (data not shown) variable expression of CD95 (Fas/APO1), a 48-kD transmembrane glycoprotein, at present identified as an important mediator in the apoptotic process mediated by NK-cells. Although this finding suggested the possible susceptibility of tumors to the action of infiltrating NK-cells, in a previous study, we concluded that the number of NK-cells that are present in the stroma of brain metastases does not correlate with the number of apoptotic tumor cells [10]. Therefore, it is possible that NK-cells do not play an important role in the immunological defense against brain metastases. The purpose of the present study is to add new data to this hypothesis, verifying if the degree of local immunological response against a metastatic brain tumor, measured by the degree of NK-cell infiltration within the tumor tissue, influences the clinical behavior, in terms of influencing the capacity of the brain to be protected for the development of new metastases or local tumor recurrence. Our present results showed that the time free of new cerebral affectation for metastatic dissemination or recurrence of the previously resected tumor is not related with the degree of immunological response mediated by the presence of NK-cells. Although it is obvious that our present series has scarce number of cases and that multiple factors, mainly the evolution of the systemic disease, can influence the clinical behavior of patients suffering metastatic brain disease, we think that our present analysis represents a new argument supporting that in brain metastases, the immune response mediated by CD57^+^ NK-cells plays a doubtful role. This consideration should be kept in mind in therapeutic trials based on the hypothetical defensive action of the NK-cells against metastatic brain tumors.

## 5. Conclusion

Our present findings suggest that clinical behavior in metastatic brain disease is not influenced by the immunological response mediated by CD57^+^ NK-cells.
